# Increase the Sanitary Inspectors

**Published:** 1899-03

**Authors:** 


### INCREASE THE SANITARY INSPECTORS.

WITH the Pan-American Exposition an assured fact, it is reasonable to presume that Buffalo will have as visitors, during the six months it is intended to keep the exposition open, thousands and thousands of strangers. These people will come here after hearing tales of the beauty of Buffalo as a home city; of its wonderfully clean streets and its health.

All these people will come here expecting to find these things true. They will. But they should find more. They should come into Buffalo and find a model city; they should find a city as nearly perfect as it is possible to make a city in this rushing, fin-de-ciecle age. Then will the fame of Buffalo be spread broadcast over the whole, wide world. Money and population will pour in and Buffalo —well, we Buffalonians are a sanguine lot and there's no knowing where we will stop when once our growing pains begin in earnest.

But to make this a model city it must be absolutely and unequivocally clean and healthy. It must be perfectly and systematically and regularly inspected and the inspection should begin at once. Not next year, but now. The city should be districted and reports on each district, detailed and exhaustive, made each month. Violators of ordinances should be relentlessly pursued and mercilessly punished.

To make Buffalo a model city of health and cleanliness, a city which would be the wonder of the world and rival even Paris, Dr.

Wende should be given authority to increase his staff of inspectors. Every new inspector should be a physician. Each physician should be chosen because of his fitness for the particular branch of inspecting he is to do. There should not be any guess work. To guess is to woo failure. Every nook and cranny in this city should be inspected and kept under inspection from now until the last visitor has left Buffalo.

For two years this would cost the city, say $20,000, and Buffalo would be a $1,000,000 richer in fame.

A full force of inspectors is not necessary just now, but the increase should be begun at once, and additional inspectors added from time to time up to the spring of 1900, when a full force should be at work and a perfect system of inspection in operation.

